# Feasibility indicators of telemedicine for patients with dementia in a public hospital in Northeast Brazil during the COVID-19 pandemic

**DOI:** 10.1371/journal.pone.0268647

**Published:** 2022-05-23

**Authors:** Danielle Pessoa Lima, Ingrid Barros Queiroz, Alexandre Henrique Silva Carneiro, Daniela Araújo Aragão Pereira, Camila Silva Castro, Antonio Brazil Viana-Júnior, Charlys Barbosa Nogueira, João Macedo Coelho Filho, Rômulo Rebouças Lôbo, Jarbas de Sá Roriz-Filho, Pedro Braga-Neto

**Affiliations:** 1 Division of Geriatrics, Department of Clinical Medicine, Universidade Federal do Ceará, Fortaleza, Brazil; 2 Medical School of Universidade de Fortaleza, Fortaleza, Brazil; 3 Division of Neurology, Department of Clinical Medicine, Universidade Federal do Ceará, Fortaleza, Brazil; 4 Clinical Research Unit of Hospital Universitário Walter Cantídio, Universidade Federal do Ceará, Fortaleza, Brazil; 5 Center of Health Sciences, Universidade Estadual do Ceará, Fortaleza, Brazil; Prince Sattam Bin Abdulaziz University, College of Applied Medical Sciences, SAUDI ARABIA

## Abstract

**Background:**

The use of telemedicine has become a fundamental tool in healthcare in recent years, especially at times of Covid-19 pandemic. Currently, there are several telemedicine tools that are simple, inexpensive, and effective means of communication. This article aims to describe indicators of feasibility including patient recruitment, attendance, discomfort (internet connection issues and/or noncompliant patient behavior), satisfaction, and travel time and cost savings of virtual telemedicine consultations for patients with dementia.

**Methods:**

The study was conducted at the Geriatrics Department of Hospital Universitário Walter Cantídio (HUWC) in Fortaleza, Brazil, between May 1^st^ and December 31, 2020. The eligibility criteria included previous diagnosis of dementia syndrome and receiving care at the hospital’s dementia outpatient clinic in face-to-face consultations in the preceding 12 months. Patients were excluded if they did not feel comfortable with virtual consultations, did not have the required communication technology available or their caregiver was not available to attend the remote consultation. The patients were recruited from the outpatient dementia clinic’s medical appointment scheduling list. The intervention was designed as a one-time consultation and it included treatment approaches and health promotion recommendations.

**Results:**

Patient recruitment, attendance and discomfort rates were 85.5%, 97.7% and 9.4%, respectively. To attend face-to-face visits, they reported an average travel time (including the consultation) of 233.21 minutes and average total cost of 60.61 reais (around USD 11). The study intervention was well accepted among the patients and their caregivers with 97.6% being satisfied. Many were happy to avoid long waits in crowded medical waiting rooms and the risk of covid-19 contagion.

**Conclusions:**

We found good recruitment, attendance, and acceptance rates of remote care for the follow-up of dementia patients as well as low discomfort rates.

**Trial registration:**

Brazilian Trial Registry (REBEC) RBR-9xs978.

## Introduction

On March 18, 2020 government’s authorities imposed social distancing measures and public gathering restrictions in the state of Ceará, Northeast Brazil, in response to the COVID-19 pandemic [1]. Elective face-to-face consultations had to be rescheduled and the need for health care during the pandemic called for telehealth solutions. National initiatives have been launched to review and update previous restrictions to telehealth practice and expand its use as a solution for improving care provided in the national state-funded health system [2]. Brazil’s Ministry of Health issued a new ordinance authorizing the practice of telemedicine in public and private settings and operationalizing measures to handle the pandemic [2].

The workup, diagnosis, and clinical follow-up of elderly with neurodegenerative diseases such as dementia may have been harmed by a shift in resources and professionals to combat the pandemic. Besides those living alone in community felt lonely due to social isolation and the absence of group activities [3]. Behavioral and psychological symptoms of dementia can be triggered or exacerbated by several risk factors (social isolation; pharmacology adherence disruption; carers’ load; reduction of nonpharmacologic techniques; absence of medical assessment; change in house routine) [4,5].

The use of telemedicine has become a fundamental tool in healthcare in recent years, especially at times of Covid-19 pandemic [6]. Currently, there are several telemedicine tools that are simple, inexpensive, and effective means of communication [7]. The merit of remote appointments is increasing the access and lowering the costs, especially if the distance or mobility is a critical factor. The most important demerit is the lack of physical examination [8]. Synchronous videoconferencing consultation is appropriate for a clinical interview, which is essential in delivering routine care for the elderly with dementia. It also allows clinicians to see the patient’s home surroundings while at a safe distance to safeguard this high-risk group from covid-19 [9]. However, the effectiveness of this form of treatment is dependent on availability to technology, as well as the capacity to see, hear, and interpret the clinical interview [10,11]. Telemedicine in the home requires the use of a smartphone, tablet, or computer, as well as a stable Internet connection and the capacity to handle technological difficulties [12]. Telemedicine needs patients or caregivers to have good hearing and vision, as well as physical and cognitive abilities, to communicate with clinicians and understand their treatment plan. These prerequisites can be difficult for older persons with dementia, who may have various demands when dealing with telemedicine systems [11]. The effectiveness of telemedicine delivery is also dependent on the technological skills of the care provider and the patient’s capacity to connect with the physician remotely. This added weight of technological skill required by telemedicine may potentially restrict the successful acceptance of telemedicine as a routine modality of geriatric care [13].

Online social networking appears to have a rising number of advantages, including increases in cognitive, social, and emotional well-being in the elderly. Executive functioning, working memory, episodic memory, perceived quality of life, social support, and engagement, as well as decreased depressive symptoms and feelings of loneliness, are all advantages [14,15].

To the best of our knowledge, no study has assessed the feasibility of using telemedicine for delivering care to patients with dementia in Brazil’s public health system [10,11,13]. Official data from the Government of the State of Ceará show that 60.9% of adults over 25 years old have not completed basic education. In addition, 40% of the population has a monthly per capita income equivalent to USD 55.75 or less [16]. This study aims to describe indicators of feasibility including patient recruitment, attendance, sense of safety and satisfaction and travel time and cost savings. The purpose was not to assess the effectiveness of the study intervention, but rather to examine the feasibility of geriatric consultations via telemedicine for delivering care to patients with dementia in the brazilian public health system.

## Methods

### Trial design

Telegeriatric is a term that derives from the junction of Tele (from Greek, meaning “at a distance”) and Geriatrics (from Greek, “relating to old people”). In our study, it represents the act of attending elderly patients using video calls by mobile devices.

We performed a single-center one-arm study to describe indicators of feasibility (including patient recruitment, attendance, acceptance, sense of safety and benefit, satisfaction, and travel time and cost savings) of virtual medical consultations.

### Study design

The study was conducted at the Geriatrics Department of Hospital Universitário Walter Cantídio (HUWC) in Fortaleza, Brazil, between May 1^st^ and December 31, 2020. It was approved by HUWC Ethics Committee (registration number 31779920.1.0000.5045) and the Brazilian Trial Registry (REBEC) RBR-9xs978. All patients or their caregivers signed an online informed consent form.

### Study participants

Participants were consecutively drawn from the dementia outpatient clinic’s medical appointment scheduling platform at HUWC. Those who met the inclusion criteria were selected until the required recruitment period (a total of eight months) was achieved. The patients were invited in a phone call to participate in the feasibility study. Those who agreed to participate were screened for eligibility and, if eligible, they were asked to attend a remote medical consultation. The WhatsApp-based intervention included video and text messages. Recent studies have emphasized broad opportunities of WhatsApp messenger as an adjunctive tool for telemedicine [17]. We chose this tool because our sample is familiar with the use of this technology in their everyday life. We used two smartphones exclusively for telegeriatric care, both with a password for unlocking and were kept in the hospital after the service. WhatsApp’s end-to-end encryption policy has increased cyber-security by ensuring that messages, photos, videos, voice messages, documents, and calls are securely transmitted in a bidirectional approach between the caller, receiver, or vice versa, making communication as private as face-to-face conversations [18].

The eligibility criteria included previous diagnosis of dementia by our outpatient clinic’s geriatric staff according to the diagnostic criteria of the Diagnostic and Statistical Manual of Mental Disorders 5th edition (DSM-5) and receiving care at the hospital’s dementia outpatient clinic in face-to-face consultations in the preceding 12 months. Patients were excluded if they did not feel comfortable with virtual consultations, did not have the required communication technology available or their caregiver was not available to attend the remote consultation. If the patient did not have a brain image to confirm the etiological diagnosis of the dementia syndrome, he or she was identified as dementia under evaluation.

### Study intervention

We reviewed medical charts before and during the consultation to check for comorbidities and medication use. The remote consultations were held once a week during the morning hours and followed an interview approach similar to the face-to-face consultations. Since the study setting was a public teaching hospital, consultations were carried out by medical residents and interns during their rotation in this service under the supervision of one geriatrician who is a hospital employee.

The remote consultations involved treatment approaches, health promotion recommendations, drug prescription, complementary diagnostic tests, and referral to other health providers. Patients were sent education materials with health recommendations and medication use schedules to improve their adherence (supplementary files 1 and 2). We developed two different education materials (PDF files), one for patients with preserved mobility and other for those with limited mobility.

**Fig 1:**
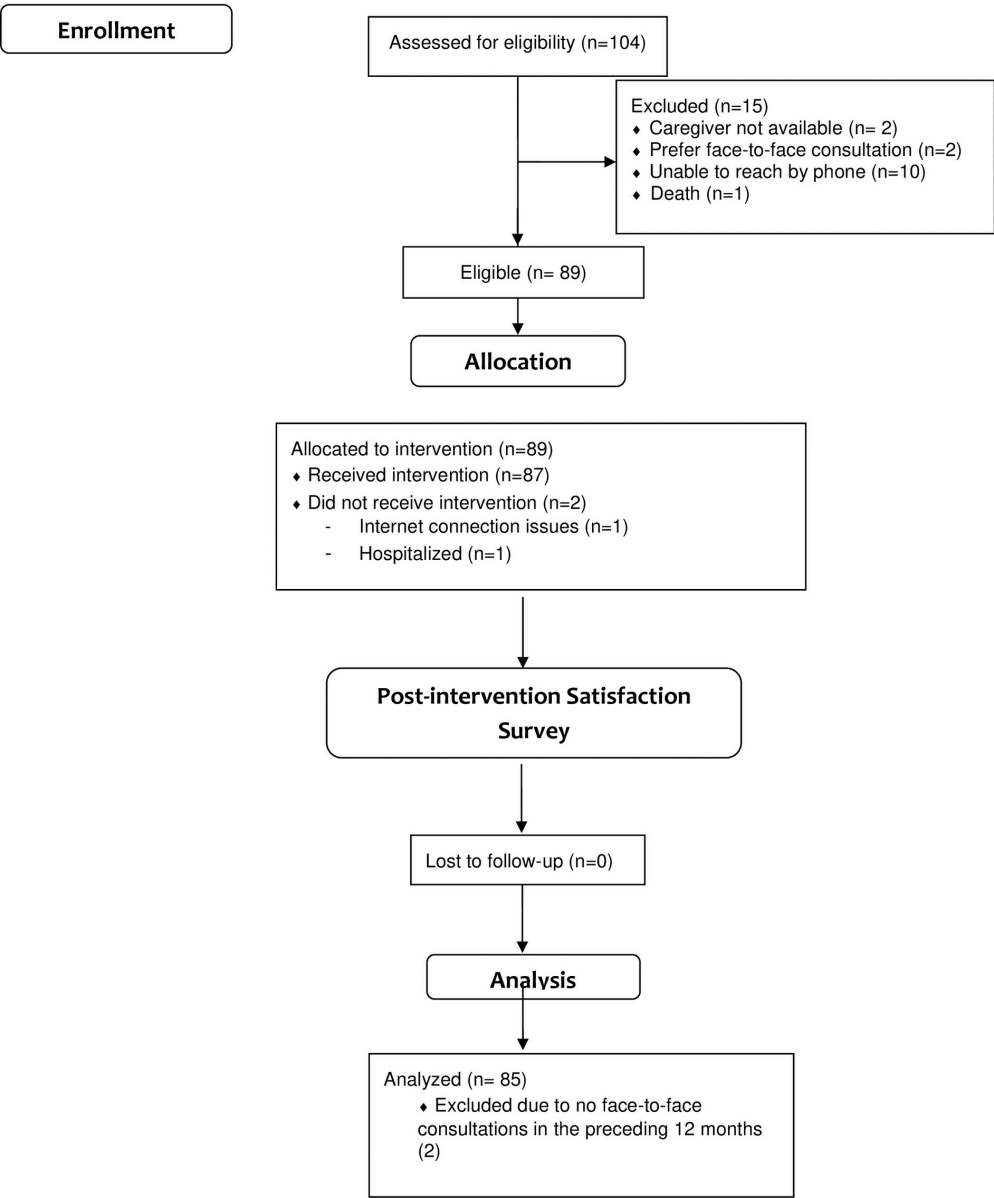
CONSORT 2010 flowchart.

The intervention was designed as a one-time consultation (see flowchart in Fig 1) before the next face-to-face consultation. Remote reassessment was determined by the attending geriatrician or at caregivers’ request. An urgent face-to-face appointment was made when a physical examination was required.

We used the Research Electronic Data Capture (REDCap) software for data collection and management.

### Sample size

The feasibility criteria that were set apriori to the trial were: recruitment and attendance rates, sense of safety and satisfaction and travel time and cost savings. The study sample was drawn consecutively among those meeting the eligibility criteria during the recruitment period from May to December 2020. The period of recruitment was established based on logistics. The HUWC coordination permitted virtual visits from May to December 2020, considering the pandemic’s social constraints and staff availability. The purpose of this study was not to assess the effectiveness of the intervention, but rather to decide whether the virtual consultations are viable in our outpatient clinic. All studies should have a sample size justification. Not all studies however need to have a sample size calculation. For pilot and feasibility trials, while a sample size justification is important, a formal sample size calculation may not be appropriate [19]. We used a “convenient” sample of our dementia outpatient clinic.

#### Characterization measures

Data was collected through medical chart review and patient questionnaires. Baseline and demographic data included age; gender; type of caregiver (formal [paid]/informal [unpaid]); dementia duration; presence of chronic comorbidities; medication use; rehabilitation care; fall events; neuropsychiatric symptoms; sleep disturbance complaints; constipation; dysphagia; and urinary incontinence. The participants were also asked about functional decline due to social restrictions during the pandemic. Duration of dementia was defined as the number of years that the patient received the diagnosis of dementia by a physician. The duration from onset of dementia symptoms was defined as the number of years that the relatives noticed the patient’s trouble in cognition.

We administered three dementia-related geriatric questionnaires: The Functional Assessment Staging Tool (FAST); the Katz Activities of Daily Living (ADL) scale; and the Abbreviated Mental Test 4 (AMT4). The FAST is easy to administer and has excellent validity for assessing functional capacity over the course of Alzheimer’s disease and it is also used in other dementias [20].

We used the AMT-4 to assess cognitive function, which is a brief cognitive test widely used, with good specificity (88%) and good reliability [21]. The four questions asked were: 1 –current time (correct answer: ±1 hour); 2 –home address instead of hospital name as the patients were at home; 3 –date of birth; 4 –counting backwards by 1 from 20 to 1.

The Katz scale is widely used to assess activities of daily living (ADLs) rated on a scale of 0 to 6. We administered a version of the Katz scale that was translated and culturally adapted to Brazilian Portuguese by Lino et al. (2008), where higher scores indicate more dependence [22].

### Recruitment

Eligible patients were asked to sign an informed consent form through an online permission form and an appointment was scheduled using WhatsApp messenger or via telephone. They were given some instructions to optimize the remote consultation: choose an adequate room with strong wi-fi signal; have their caregiver or a family member present during the entire appointment; and have their most recent test results and list of medications at hand.

The recruitment rate for telemedicine consultations was calculated as the number of patients agreeing to participate in the study divided by total number of patients which we have attempted to contact.

### Attendance

The attendance rate was calculated as the proportion of virtual medical consultations completed as scheduled. The intervention was considered feasible if at least 80% of the consultations could be completed [23].

### Acceptance

A self-administered questionnaire was used to evaluate whether our telemedicine intervention was feasible, safe, satisfying, and well-accepted for future use. This questionnaire was answered with the help of the caregiver for patients with early dementia and by the caregivers themselves for patients with moderate to severe dementia. They were asked to state the identity of the respondent. The questionnaire consisted of 12 questions with a visual analogue scale (VAS) to evaluate satisfaction (worst on the left end and best on the right end) and one additional open-ended question. The participants were asked to mark the point (face) on a horizontal line that was representative of their level of satisfaction. For each question, they could select only one option.

The questionnaire has five domains: feasibility (items 1–2); sense of safety (items 3–5); appreciation and satisfaction (items 6–7); effectiveness (items 8–9); acceptance and future use of the intervention (items 10–12). Lower scores indicated lower perceived interest, safety, satisfaction, acceptance, and benefits of telemedicine. We also included an open-ended question for suggestions and comments. The VAS scale was sent on WhatsApp as a Google Drive link. This same questionnaire has been administered by other authors [24,25]. If the participants did not complete the questionnaire within 45 days, it was administered through a phone interview with the participants’ caregivers.

### Discomfort

Patient discomfort during consultation was assessed by the number of consultations with internet connection issues and/or the interviewer’s perception of noncompliant patient behavior divided by the number of consultations.

### Travel time and cost savings

Information on travel time and costs was collected during the interview to capture both time and financial burden of face-to-face consultations. They were asked about transportation, average travel time from the clinic to their home, and travel expenses.

### Statistical analysis

For numerical variables, data was presented as means, standard deviations and medians. For categorical variables, data were described as frequencies.

## Results

We recruited a total of 104 patients from the geriatric outpatient clinic’s medical appointment scheduling platform; 89 agreed to participate in the study. The recruitment rate was 89/104 x 100, i.e., 85.5%. The attendance rate was 87/89 x 100, i.e., 97.7%. We had two dropouts because two patients were not able to attend the scheduled consultation, one due to internet connection issues and the other one was hospitalized.

The telemedicine team evaluated a total of 4 patients every Tuesday morning, except holidays. Remote reassessments were requested for 20 (12.9%) patients. The reasons for reassessment included medical complications (2 patients); behavior disturbances (4 patients); both clinical and behavior reasons (1 patient); medication withdrawal (1 patient); complementary test results (1 patient); and family request (2). Six (7.0%) patients were advised to come to the clinic as early as possible for a face-to-face consultation as they required physical examination.

Discomfort was evidenced during the telemedicine consultation for 8 (9.4%) patients due to internet connection issues (7 patients) and noncompliant behavior (1 patient). Fig 1 shows the study flowchart. Table 1 summarizes the demographic characteristics of our sample.

**Table 1:** Baseline demographic characteristics of the study participants.

Variables	Values
Gender	
Male	24 (28.2%)
Female	61 (71.8%)
Age	81 ± 12 (83)
Education	
≤ 8 years	60 (70.6%)
>8 years	25 (29.4%)
Marital status	
Living with a partner	35 (41.2%)
Living without a partner	50 (58.8%)

Data expressed in frequency (percentage) or mean ± standard deviation (median).

Eighty-seven patients attended remote geriatric consultations in this study; however, two were excluded from the analysis because they had not had any consultations in the 12 months preceding the study. The final sample included 85 participants. The mean age was 81 ± 12 years old, mean dementia duration of 5.3 ± 3.2 years and mean duration from onset of dementia-related symptoms of 7.5 ± 3.8 years. Most participants were female (71.8%), 37.6% had Alzheimer’s disease and 14.1% mixed dementia (Alzheimer’s disease and vascular dementia). The main comorbidities included systemic arterial hypertension (72.9%); dyslipidemia (50.6%); osteoporosis (36.4%); and diabetes mellitus (34.1%). The most used classes of drugs were antihypertensive drugs (67%); antidepressants (51.8%); vitamin D supplements (49.4%); statins (40%); calcium supplementation (31.8%); oral antidiabetic drugs (30.6%); and atypical antipsychotic drugs (27.1%).

Over half of the participants (N = 60; 70.58%) had less than 8 years of schooling and the caregiving role was played by a family member for the large majority (90.4%). The average number of medications used was 7 ± 3.19. Thirty-seven (44%) patients showed behavior changes complaints and 33 (39.8%) had sleep disturbance complaints. Functional decline during the pandemic was reported for 23 (28.4%) patients. The median disability index of ADLs assessed by the Katz scale was 4. Table 2 shows dementia-related clinical information of the participants and the Functional Assessment Staging Tool of study participants. Table 3 summarizes the participants’ medical conditions.

**Table 2:** Dementia-related medical characteristics and functional assessment staging tool of study participants.

Variables	Values
Reason for dementia consultation	
*Alzheimer’s disease*	32 (37.6%)
*Vascular dementia*	3 (3.5%)
*Mixed dementia (Alzheimer’s disease and vascular dementia)*	12 (14.1%)
*Lewy body dementia*	3 (3.5%)
*Parkinson’s disease dementia*	2 (2.4%)
*Dementia under evaluation*	30 (35.3%)
*Others*	3 (3.5%)
KATZ INDEX	3.27 ± 2.32 (4)
*Functional Assessment Staging Tool (FAST Scale)*	
*(4) Mild dementia*	27 (31.7%)
*(5) Moderate*	2 (2.4%)
*(6) Moderately severe dementia*	2 (2.4%)
*(7) Severe dementia*	7 (8.4%)
AMT-4 SCORE	1.34 ± 1.53 (1)

Data expressed in frequency (percentage) or mean ± standard deviation (median).

**Table 3:** Medical conditions of the study participants.

Variables	Values
Number of medications used	7 ± 3.2 (7)
Behavior changes complaints	37 (44%)
Sleep disturbance complaints	33 (39.8%)
Intestinal constipation complaints	22 (25.9%)
Dysphagia complaints	27 (31.8%)
Urinary incontinence complaints	58 (68.2%)
Functional decline during the COVID-19 pandemic	23 (28.4%)
Fall events in the preceding 6 months	21 (25.3%)
Physical therapy rehabilitation during the COVID-19 pandemic	12 (14.1%)
Physical activity for at least 30 minutes 3 times per week	6 (7%)
Went out in the last month during the COVID-19 pandemic	37 (43.5%)
Anticholinesterase drug use	54 (63.5%)
Memantine use	42 (49.4%)
Vaccinated for H1N1 and flu this year	84 (98.8%)

Data expressed in frequency (percentage) or mean ± standard deviation (median).

The most frequently medical prescriptions were for the treatment of osteoporosis and osteopenia including bisphosphonates; denosumab; vitamin D and calcium supplements (71.4%); requests of laboratory tests (60.7%); physical rehabilitation care (33.3%); bone densitometry (26.2%); laxative drugs (17.9%); and medication for controlling behavior changes (17.9%). The average duration of WhatsApp video call including medical consultation, health promotion recommendations and questionnaire administration was 46 minutes (SD 18).

For attending face-to-face consultations, the most common types of transportation used were the patient’s own car (54.1%); Uber (27.1%); taxi (11.8%); and bus (8.2%). Most patients lived in the metropolitan area of Fortaleza (91.8%) and only 7 patients (8.2%) traveled from other cities within the state of Ceará. Regarding the caregiver’s social burden, 34.5% missed work to attend face-to-face consultations. Table 4 shows information on travel time and expenses associated with face-to-face consultations in the same service. The highest total expense with face-to-face consultation was 460 reais (i.e., 44% of the current minimum wage per month in Brazil).

**Table 4:** Financial and travel time burden associated with face-to-face consultations.

Question	Mean	SD	Median
How long (in minutes) does it take your round-trip to our clinic?	55.25	60.67	40
How much do you spend (in reais*) on round-trip transportation to get to our clinic?	35.31	44.64	20
How much do you spend (in reais*) on food while waiting for your appointment at our clinic?	6.86	15.87	0
How much (in reais*) was your and/or your caregiver’s earning loss associated with your appointment at our clinic?	19.41	38.26	0
How much time (total in minutes) do you spend in terms of travel (round-trip) and consultation at our clinic?	233.21	117.58	240
How much (in reais*) do you and/or your caregiver spend in total to attend an appointment at our clinic?	60.61	76.01	31

*Brazilian currency. Abbreviations: SD: Standard deviation.

**Fig 2:**
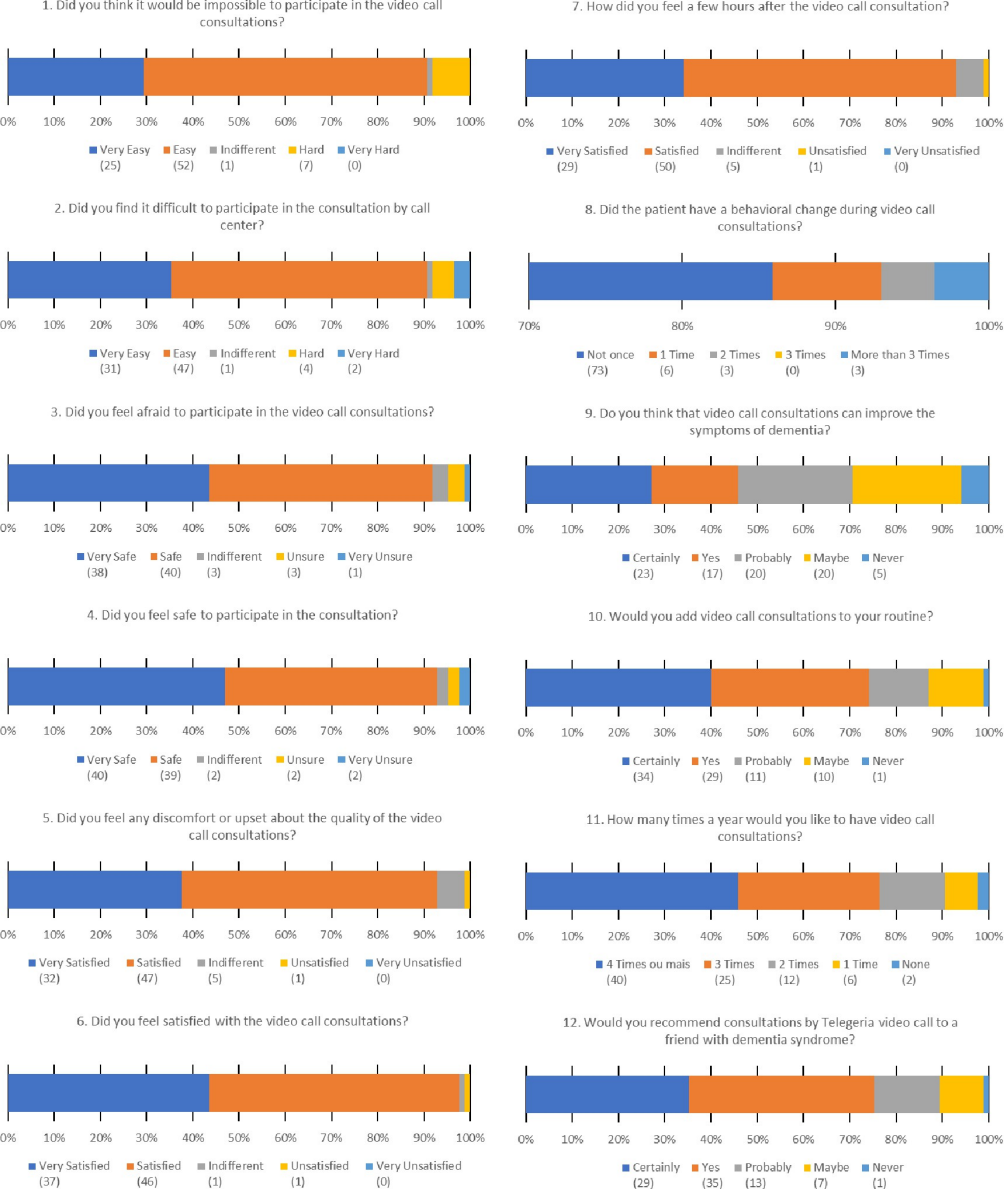
Patient satisfaction survey.

Overall, the telemedicine approach was well-accepted among the participants as assessed by the acceptance domain of VAS for satisfaction, which was greater than 90% (satisfaction was the top-rated item). The worst rated question was that related to improvement of dementia symptoms with the intervention, with about 45% of the respondents stating that they believed remote consultations could help improve dementia symptoms (Fig 2).

The answers to an open-ended question for suggestions, claims and comments of the VAS are described in Table 5.

**Table 5:** Advantages, disadvantages, and suggestions of medical consultations online–telegeriatrics.

Advantages	Values
Shorter time interval between appointments	24%
Faster response time for problem solving	12%
Not having to move the patient to the appointment	12%
Getting prescriptions without having to go to the clinic	9%
**Disadvantages**	**Values**
Longer medical appointments duration	15%
Bad internet connection	12%
**Suggestions**	
Offer an agenda with more available times to schedule the appointment	
Keep the teleconsultation and alternate with face-to-face consultations	

Frequency of answers for each question in parenthesis.

## Discussion

We found that 51.7% of the study participants had Alzheimer’s dementia, either alone or mixed dementia with cerebrovascular disease. The large proportion of patients with dementia under evaluation in our sample is because we were not able to assess brain imaging studies and/or laboratory tests to confirm the etiological diagnosis. Public patients endure long delays for diagnostic tests and imaging studies.

The participants were mostly female and this finding is likely due to women’s greater longevity as risk of developing dementia increases with age [26]. They were also less educated than their counterparts in developed countries with 18.8% illiterate and 51.8% with 1–7 years of schooling [27]. Neuropsychiatric symptoms including delusion, agitation, aggression, emotional lability, apathy and aberrant motor behavior were reported for 44% in the preceding month. Studies have showed that the prevalence of neuropsychiatric symptoms varies between the different types of dementia and stages [28,29]. Sleep disturbances in the preceding month were reported for 39.8% of the study participants. Sleep and circadian disturbances are common in all types of dementia affecting the patient’s quality of life and increasing caregivers’ burden [30].

Functional decline was perceived by 28.4% of the caregivers. Only 14.1% of the sample was attending physical therapy rehabilitation sessions during the COVID-19 pandemic; and 93% were not engaging in any physical activity for at least 30 minutes 3 times per week.

Older people are most vulnerable to the impact of confinement and they experience worsening of their medical conditions [31]. The World Health Organization (WHO) warned that people with cognitive impairments and dementia face a greater challenge during the pandemic [32]. They clearly have difficulty understanding the current public health situation and are unable to comply with health measures such as staying at home and wearing a mask [31]. In addition, discontinuation of face-to-face activities including rehabilitation sessions, medical appointments and programs at daycare centers has aggravated cognition and functioning of this population. Reduced support available to caregivers has increased their burden [3]. Europe’s Alzheimer’s Society stresses that it is helpful for patients with dementia to build a good support network, keep well informed, ensure food and medical supplies, remain physically and mentally active, and stay socially connected [32].

In contrast with large studies [33–36]. carried out in urban and rural settings, we found lower financial and travel time burden because our sample consisted mostly of people living in a metropolitan area. Another reason explaining lower total expenses in our sample is that almost all caregivers were family members either unemployed or who took paid time off work (as Brazilian legislation permits) to take their relatives to medical appointments. In Brazil, family members take on the caregiving role because most families cannot afford formal (paid) caregivers. A 2018 study evaluating direct and indirect costs of dementia in Brazil showed that total indirect costs per month (USD 843.63) were higher than their reported per capita household income (USD 286.41). Indirect costs involve caregivers’ loss of productivity and time spent in patient support [37].

The study had a small qualitative part in the satisfaction questionnaire. The responses to the one open-ended question were evaluated individually and grouped using repeated meanings. We examined the answers to the open-ended question in the satisfaction survey to better understand our sample’s experience with this new approach. The main disadvantage of telemedicine was inability to perform physical examinations. In later face-to-face encounters some caregivers admitted giving lower satisfaction ratings for fear the clinic will stop face-to-face consultations. Their main suggestion was to schedule video consultations more frequently with more convenient flexible hours because video consultations in the study were limited to one shift per week.

The main advantage of the telemedicine intervention was to deliver care to the patient at home. Our hospital (study site) is in a busy area providing service to a broad population, so caregivers often encounter heavy traffic and difficult transport conditions to bring their patients (bedridden or in a wheelchair) for consultations, which is costly and time-consuming. Given the high prevalence of neuropsychiatric symptoms in patients with dementia syndrome, they usually experience distress when removed from familiar environments. Another important advantage stressed is avoiding exposure of older patients to the coronavirus in crowded waiting rooms as they are most vulnerable to severe forms of COVID-19.

The question that ranked the lowest was: “Do you think video consultations can help improve dementia symptoms?” A possible explanation is that it was not understood as originally intended. This question was designed to assess additional dementia symptoms such as agitation, insomnia, or depression. However, the question was vague and gave rise to a different interpretation, i.e., full resolution of symptoms after a single video consultation. Another possible reason is that rehabilitation care prescribed during the remote consultation was often not provided to the patients due to few public rehabilitation centers being available as well as mobility constraints requiring home care. Home care rehabilitation services are seldom provided in a public setting in Northeast Brazil [38]. In addition, the intervention involved only care delivered by a single physician, though dementia management requires a multidisciplinary approach [37].

We chose to deliver care through telemedicine only to patients who attended our outpatient clinic in the preceding year. First, to ensure the patients underwent thorough assessment and proper diagnosis, and that caregivers and family members knew how to handle test requests and prescriptions of costly medication such as anticholinesterase drugs available through the hospital.

Recruitment and attendance rates in our study were high and are consistent with the findings of previous studies that showed that telemedicine consultations for monitoring chronic conditions are a convenient approach because patients avoid traveling to the medical facility for routine follow-up appointments and they are more convenient and safer for patients and less burdensome to caregivers [35–39]. Telemedicine can also be used for dementia assessments. Previous studies have demonstrated the feasibility of remote administration of assessment scales and tests to patients with Alzheimer’s disease and their caregivers as well as the feasibility of administration of The Mini-Mental State Examination [40], The Geriatric Depression Scale [41], Montreal Cognitive Assessment [42], Caregiver’s Burden Scale [41], The Revised Memory and Behavior Problems Checklist [41].

Most previous studies of telemedicine consultations involved patients who were more educated and familiar with the use of internet than those in our sample [6,8,23,34,43]. Therefore, we chose to use WhatsApp as a communication technology tool because they would find it easier to use than other platforms. Given the popularity, availability, portability and technological capabilities of smartphones, eHealth has an enormous potential and can positively impact chronic disease management [44,45]. A recent clinical trial (2020) conducted in Hong Kong found that only the telehealth through videoconferencing applications on mobile devices including Zoom, WhatsApp, and FaceTime compared with phone conversations alone during a period of social distancing due to the COVID-19 pandemic was associated with a positive impact on the quality of life among community elderly residents with neurocognitive impairments as well as on the quality of life and perceived burden of their caregivers [46].

To the best of our knowledge this is the first study conducted in Brazil to assess the feasibility and acceptance of a telemedicine approach for patients with dementia using WhatsApp. Management of patients with dementia is quite challenging as it involves several multidisciplinary providers and dedicated caregivers [47] Health care expenditures are significantly high including personal and government spending [37]. According to Instituto Brasileiro de Geografia e Estatística (IBGE), the national agency responsible for official collection of statistical and geographic information, Brazil is the largest Latin America country with around 210 million people and nearly 30.1 million elderly accounting for 14.3% of the population, which poses a heavy challenge to the national health system which is predominantly publicly funded [48].

This study showed good patient satisfaction with telemedicine consultations for patients with dementia, reduced travel and financial burden and increased patient safety as caregivers do not have to deal with stressful situations in waiting rooms for face-to-face consultations and the risk of coronavirus contagion during the COVID-19 pandemic. The participating resident doctors were also satisfied that a telemedicine approach is feasible for geriatric monitoring of patients in a public setting during a challenging period of social distancing restrictions.

The main limitations of this study were the small sample size and the absence of a control group attending face-to-face consultations. The main purpose of our study was to describe indicators of feasibility, patient satisfaction, benefit impression, and travel time and cost savings, so we did not collect data on effectiveness. A large multicenter study is needed to further examine the application and effectiveness of this intervention. We studied a sample that was mostly urban, but underserved populations (e.g., from rural areas) may benefit more from this intervention. Feasibility indicators should be assessed for patients in remote localities and rural areas.

## Conclusions

Our research found that, despite serving in a region with low education rates and per capita income, there is good acceptance and appreciation of distance care for the follow-up of dementia patients in our sample. However, the efficacy and safety of telemedicine treatments for dementia patients must be studied further in big prospective trials.

## Supporting information

S1 Checklist TREND statement checklist. (DOCX)

S1 File (DOCX)
