# Supplementary material for: Feasibility indicators of telemedicine for patients with dementia in a public hospital in Northeast Brazil during the COVID-19 pandemic
Source: PLoS One. 2022 May 23;17(5):e0268647. doi: 10.1371/journal.pone.0268647 (PMC9126396; doi:10.1371/journal.pone.0268647)
Supplement: S1 File — (DOCX) [file pone.0268647.s002.docx]

**Title page**

**Title:** Telemedicine for delivering care for patients with dementia at a tertiary hospital in Northeast Brazil: impact of telegeriatrics during the COVID-19 pandemic (TeleGeriatrics Protocol)

**Authors:** Danielle Pessoa Lima^1,2^**,** Ingrid Barros Queiroz^1^**,** Alexandre Henrique Silva Carneiro^1^, Daniela Araújo Aragão Pereira^1^, Camila Silva Castro^1^, Antonio Brazil Viana-Júnior^4^, Charlys Barbosa Nogueira^1^, João Macedo Coelho Filho^1^, Rômulo Rebouças Lôbo^1,3^, Jarbas de Sá Roriz-Filho^1^, Pedro Braga-Neto^4,5^.

^1^Division of Geriatrics, Department of Clinical Medicine, Universidade Federal do Ceará, Fortaleza, Brazil

^2^Medical School of Universidade de Fortaleza, Fortaleza, Brazil

^3^Clinical Research Unit of Hospital Universitário Walter Cantídio, Universidade Federal do Ceará, Fortaleza, Brazil

^4^Division of Neurology, Department of Clinical Medicine, Universidade Federal do Ceará, Fortaleza, Brazil

^5^Center of Health Sciences, Universidade Estadual do Ceará, Fortaleza, Brazil

**Corresponding author:**

Danielle Pessoa Lima

Professor of Geriatrics, Universidade de Fortaleza

Geriatrician at Hospital Universitário Walter Cantidio, Universidade Federal do Ceará

Rua Pastor Samuel Munguba, 1290 - Rodolfo Teófilo - CEP 60430-370 - Fortaleza–CE, Brazil (Campus do Porangabuçu)

Telephone number: +55 85 3366 8382

Email: dra.daniellelima@gmail.com

**Setting:** Hospital Universitário Walter Cantidio, Universidade Federal do Ceará, Fortaleza, Brazil

**Funding:** none

Author’s name, affiliation and email:

1. Corresponding author and first author:

Danielle Pessoa Lima, Physician, Master Degree

Division of Geriatrics, Department of Clinical Medicine, Hospital Universitário Walter Cantidio da Universidade Federal do Ceará (UFC), Fortaleza, Brazil;

Medical School of Universidade de Fortaleza (UNIFOR), Fortaleza, Brazil.

dra.daniellelima@gmail.com

ORCID ID: 0000-0003-4591-727X

2. Co-authors

Ingrid Barros Queiroz, Physician, Resident of Geriatrics

Division of Geriatrics, Department of Clinical Medicine, Universidade Federal do Ceará, Fortaleza, Brazil.

[dra.ingrid.bq@hotmail.com](mailto:dra.ingrid.bq@hotmail.com)

ORCID ID: 0000-0003-2888-8729

Alexandre Henrique Silva Carneiro, Physician, Resident of Geriatrics

Division of Geriatrics, Department of Clinical Medicine, Universidade Federal do Ceará, Fortaleza, Brazil.

alexandre.carneiro.geriatria@gmail.com

ORCID ID: 0000-0001-9658-4730

Daniela Araújo Aragão Pereira, Physician, Resident of Geriatrics

Division of Geriatrics, Department of Clinical Medicine, Universidade Federal do Ceará, Fortaleza, Brazil.

[pereira.a.daniela@gmail.com](mailto:pereira.a.daniela@gmail.com)

ORCID ID: 0000-0002-7345-149X

Camila Silva Castro, Physician, Resident of Geriatrics

Division of Geriatrics, Department of Clinical Medicine, Universidade Federal do Ceará, Fortaleza, Brazil.

[camilascastromd14@gmail.com](mailto:camilascastromd14@gmail.com)

ORCID ID: 0000-0002-8375-6705

Antonio Brazil Viana Júnior, Statician, Master Degree

Clinical Research Unit of Hospital Universitário Walter Cantidio, Universidade Federal do Ceará, Fortaleza, Brazil.

brazil.estatistico@gmail.com

ORCID ID: 0000-0001-6608-3134

Charlys Barbosa Nogueira, Physician, PhD

Division of Geriatrics, Department of Clinical Medicine, Universidade Federal do Ceará, Fortaleza, Brazil.

ORCID ID: 0000-0003-0951-4748

João Macedo Coelho Filho, Physician, PhD

Division of Geriatrics, Department of Clinical Medicine, Universidade Federal do Ceará, Fortaleza, Brazil.

[jmacedocoelho@ufc.br](mailto:jmacedocoelho@ufc.br)

ORCID ID: 0000-0001-6608-3134

Rômulo Rebouças Lôbo, Physician, PhD

Division of Geriatrics, Department of Clinical Medicine, Universidade Federal do Ceará, Fortaleza, Brazil

Jarbas de Sá Roriz Filho, Physician, PhD

Division of Geriatrics, Department of Clinical Medicine, Universidade Federal do Ceará, Fortaleza, Brazil

jarbasroriz@gmail.com

[ORCID ID: 0000-0002-5928-0399](https://orcid.org/0000-0002-5928-0399)

Senior Pedro Braga Neto, Physician, PhD

Division of Neurology, Department of Clinical Medicine, Universidade Federal do Ceará, Fortaleza, Brazil.

Center of Health Sciences, Universidade Estadual do Ceará, Fortaleza, Brazil.

[pbraganeto@ufc.br](mailto:pbraganeto@ufc.br)

ORCID ID:  0000-0001-9186-9243

**Abstract**

**Background:** Remote access to health care optimizes the use of local resources, facilitates access to care and minimizes the risk of covid-19 contagion. This study aims to describe indicators of feasibility including patient recruitment, attendance, discomfort, satisfaction and travel time and cost savings of virtual telemedicine consultations for patients with dementia. **Methods:** Virtual telemedicine consultation with patients and their caregivers will be similar to a routine face-to-face consultation. We will perform a single-center one-arm study with indicators of feasibility as the study primary endpoints.

**Ethics and dissemination:** The study is approved by the Research Ethics Committee of Hospital Universitário Walter Cantidio and all participants will provide their written informed consent (register number 31779920.1.0000.5045).

Trial results will be disseminated via peer reviewed journal articles and conference presentations, reports for organisations involved with PD and for participants. Trial registration number Registro Brasileiro de Ensaios Clínicos Registry (RBR-9xs978).

**Keywords**

telemedicine, dementia, feasibility studies

**Bullet points:**

- This is a feasibility study of a telemedicine approach for patients with dementia attending a public health care center in Northeast Brazil.
- The telemedicine intervention may be a good strategy for avoiding a stressful situation of long waits in medical rooms for face-to-face consultations and the risk of coronavirus contagion in the current COVID-19 pandemic.
- Telemedicine consultations for delivering care for patients with dementia may be feasible and effective in Brazil’s public health system.

**Telemedicine for delivering care for patients with dementia at a tertiary hospital in Northeast Brazil: impact of telegeriatrics during the COVID-19 pandemic (TeleGeriatrics Protocol)**

**Background and rationale**

On March 18, 2020 government’s authorities imposed social distancing measures and public gathering restrictions in the state of Ceará, Northeast Brazil, in response to the COVID-19 pandemic.^1^ Elective face-to-face consultations had to be rescheduled and the need for health care during the pandemic called for telehealth solutions. National initiatives have been recently launched to review and update previous restrictions to telehealth practice and expand its use as a solution for improving care provided in the national state-funded health system.^2^ Brazil’s Ministry of Health issued a new ordinance authorizing the practice of telemedicine in public and private settings and operationalizing measures to handle the pandemic.

Brazil’s aging population has grown at a fast rate in recent decades.^3^ There has been also an increase in the prevalence of neurodegenerative and neuropsychiatric diseases, especially dementias. Social and economic consequences of dementias are profound in the Brazilian population.^4^ Neuropsychiatric symptoms of dementia are common and they are often distressing for patients and caregivers. Neuropsychiatric presentations appear in various types of dementia and at different stages, and symptom frequencies and presentation may vary.^5^ Given the enormous burden of dementia for patients and caregivers, in addition to the fact that older age is associated with more multimorbidity and clinical complications requiring monitoring and control, ongoing individualized approaches are imperative for the management of this vulnerable population group.^6^

Many patients with neurodegenerative disease, particularly those living in rural areas, can likely benefit from improved health care access via telemedicine in both the short- and the long-term.^7^ Distance, functional disability, uneven geographic distribution of health care professionals, lack of social support, work constraints, travel cost and associated risks can be barriers to access of older people to specialized care.^8,9^ Remote access to health care optimizes the use of local resources, facilitates access to care and minimizes the risk of covid-19 contagion.^10^ To the best of our knowledge, no study has assessed the feasibility of using telemedicine for delivering care to patients with dementia in Brazil’s public health system. This study aims to describe indicators of feasibility including patient recruitment, attendance, sense of safety and satisfaction and travel time and cost savings. The purpose is to examine the feasibility of geriatric consultations via telemedicine for delivering care to patients with dementia in the public health system.

Hypothesis

We hypotesize that TeleGeriatrics will have good indicators of feasibility of geriatric consultations via telemedicine for delivering care to patients with dementia in the public health system.

SPECIFIC OBJECTIVES

Primary objective

To determine the feasibility of a telehealth video consultation using WhatsApp© for delivering care directly at home to patients with dementia in Brazil’s public health system.

Secondary objectives

To ascertain the perceived impact of covid pandemia worsening their medical conditions

**Methods/Trial design**

*Study design*

We will perform a single-center one-arm study to describe indicators of feasibility (including patient recruitment, attendance, acceptance, sense of safety satisfaction and travel time and cost savings) of virtual medical consultations. Elegible patients will be recruited from outpatient clinic’s medical appointment scheduling list.

The flow diagram of the protocol is detailed in figure 1. The study protocol was described according to the Standard Protocol Items: Recommendations

for Interventions Trials statement (SPIRIT)^11^.

Study Setting

The study will be conducted at the Geriatrics Department of Hospital Universitário Walter Cantídio (HUWC) in Fortaleza, Brazil, between May 1^st^ and December 31, 2020. It was approved by HUWC Ethics Committee (registration number 31779920.1.0000.5045) and the Brazilian Trial Registry (REBEC) RBR-9xs978. All patients or their caregivers will sign a written informed consent form.

The Hospital Universitário Walter Cantídio (HUWC) of Universidade Federal do Ceará (UFC) is a reference centre for training human resources and developing

health research. Being integrated with the Public Health System, it also acts as an important centre for healthcare in Ceará. As a reference centre for teaching, it serves as an internship field for undergraduate and graduate students of UFC’s Medicine, Nursing, Physiotherapy and Pharmacology courses.

*Study participants*

Participants will be consecutively recruited from the dementia outpatient clinic’s medical appointment scheduling platform at HUWC. Those who will meet the inclusion criteria will be selected until the required recruitment period (a total of eight months) is achieved. The patients were will be invited in a phone call to participate in the feasibility study. Those who agree to participate will be screened for eligibility and, if eligible, they will be asked to attend a remote medical consultation. The WhatsApp-based intervention includes video and text messages. Recent studies have emphasized broad opportunities of WhatsApp messenger as an adjunctive tool for telemedicine.^12^ We chose this tool because our sample is familiar with the use of this technology in their everyday life.

Eligibility criteria

The eligibility criteria include previous diagnosis of dementia syndrome according to the diagnostic criteria of the Diagnostic and Statistical Manual of Mental Disorders 5th edition (DSM-5) and receiving care at the hospital’s dementia outpatient clinic in face-to-face consultations in the preceding 12 months. Patients will be excluded if they do not feel comfortable with virtual consultations, do not have the required communication technology available or their caregiver is not available to attend the remote consultation.

*Study intervention*

The team will review medical charts before and during the consultation to check for comorbidities and medication use. The remote consultations will follow the order of patients in the waiting list; they will be conducted once a week during morning hours following an interview procedure similar to face-to-face consultations. Since the study setting is a public teaching hospital, consultations will be carried out by medical residents and interns during their rotation in this service under the supervision of one geriatrician who is a hospital employee.

The remote consultations will involve treatment approaches, health promotion recommendations, drug prescription, complementary diagnostic tests and referral to other health providers. Patients will be sent education materials with health recommendations and medication use schedules to improve their adherence (supplementary files 1 and 2). We developed two different education materials (PDF files). Patients with preserved mobility received education materials including recommendations on healthy eating, sleep hygiene, physical activity, fall prevention and non-pharmacological approaches for urinary incontinence. Those with limited mobility received materials with recommendations for healthy nutrition, sleep hygiene, approaches to reduce the risk of aspiration pneumonia and prevent skin lesions, contractures and pain.

The intervention is designed as a one-time consultation (see flowchart in Figure 1) before the next face-to-face consultation. Remote reassessment will be determined by the attending geriatrician or at caregivers’ request. An urgent face-to-face appointment will be provided when a physical examination is required.

We will use the Research Electronic Data Capture (REDCap)^13^ software for data collection and management.

Criteria for discontinuation

If there is suspicious that the patient doesn’t have dementia diagnosis or if the patient does not have a face-to-face consultation in the prior 12 months, the virtual appointment will be concluded but the patient will not be included in the analysis and the outcomes measures will not be collected.

*Sample size*

The study sample was drawn consecutively among those meeting the eligibility criteria during the recruitment period from May to December 2020. The period of recruitment was established based on logistics and financial considerations.

*Characterization measures*

Data will be collected through medical chart review and patient questionnaires. Baseline and demographic data include age; gender; type of caregiver (formal [paid]/informal [unpaid]); dementia duration; presence of chronic comorbidities; medication use; rehabilitation care; fall events; neuropsychiatric symptoms; sleep disturbance complaints; constipation; dysphagia; and urinary incontinence. The participants will also be asked about functional decline due to social restrictions during the pandemic.

We will administer three dementia-related geriatric questionnaires: The Functional Assessment Staging Tool (FAST); the Katz Activities of Daily Living (ADL) scale; and the Abbreviated Mental Test 4 (AMT4). The FAST is easy to administer and has excellent validity for assessing functional capacity over the course of Alzheimer’s disease and it is also used in other dementias^14^. It assesses a continuum over 16 successive stages, from normality through more severe dementia stages.^15^ The FAST scale has 7 stages: 1, normal cognition; 2, subjective cognitive impairment; 3, mild cognitive impairment; 4, mild dementia; 5, moderate dementia; 6, moderately severe dementia; and 7, severe dementia. Stages 6 and 7 are further subdivided into substages: substages 6a to 6e, moderately severe dementia; substages 7a to 7c, severe dementia; and substages 7d to 7g, very severe dementia.^16^ All patients in our study were FAST stages 4 to 7.

We will use the AMT-4 to assess cognitive function. Brief cognitive tests such as the AMT-4 and AMT-10 have been widely used in acute care units of UK hospitals and shown good sensitivity for detecting delirium as well as previously diagnosed dementia.^17^ The AMT-10 was not superior to the AMT-4 in a validation study conducted in Brazil.^18^ The cut-off points of three or more correct answers (AMT-4) showed an even higher sensitivity (80%) than the ten-item test, with good specificity (88%) and good reliability^19^. The four questions that will be asked are: 1 – current time (correct answer: ±1 hour); 2 – home address instead of hospital name as the patients were at home; 3 – date of birth; 4 – counting backwards by 1 from 20 to 1.

The Katz scale is widely used to assess activities of daily living (ADLs) rated on a scale of 0 to 6. It has been developed as a predictor of treatment outcomes in older people and patients with chronic conditions. This 6-item scale grades performance on self-care activities following a hierarchy of complexity: feeding; continence; transferring; personal hygiene; dressing; and bathing. Independence means being able to perform these activities without supervision, guidance or active personal assistance. The assessment is based on actual performance, not skills. Those refusing to perform an activity are considered dependent, even though they are believed to be capable of doing it. We administered a version of the Katz scale that was translated and culturally adapted to Brazilian Portuguese by Lino et al. (2008), where higher scores indicate more dependence.^20^

Primary outcome measures

*Recruitment rate*

Eligible patients will be asked to sign an informed consent form and an appointment will be scheduled using WhatsApp messenger or via telephone. They will receive some instructions to optimize the remote consultation: choose an adequate room with strong wi-fi signal; have their caregiver or a family member present during the entire appointment; and have their most recent test results and list of medications at hand.

The recruitment rate for telemedicine consultations will be calculated by the number of patients agreeing to participate in the study divided by total number of patients which we will have attempted to contact.

*Attendance*

The attendance rate will be calculated by the proportion of virtual medical consultations completed as scheduled. The intervention will be considered feasible if at least 80% of the consultations could be completed.^21^

*Acceptance*

A self-administered questionnaire will be used to evaluate whether our telemedicine intervention is feasible, safe, satisfying and well-accepted for future use. This questionnaire will be answered with the help of the caregiver for patients with early dementia and by the caregivers themselves for patients with moderate to severe dementia. They will be asked to inform the questionnaire’s respondent. The questionnaire consists of 12 questions with a visual analogue scale (VAS) to evaluate satisfaction (worst on the left end and best on the right end) and one additional open-ended question. The participants will be asked to mark the point (face) on a horizontal line that is representative of their level of satisfaction. For each question, they can select only one option.

The questionnaire has five domains: feasibility (items 1–2); sense of safety (items 3–5); appreciation and satisfaction (items 6–7); effectiveness (items 8–9); acceptance and future use of the intervention (items 10–12). Lower scores indicate lower perceived interest, safety, satisfaction, acceptance and benefits of telemedicine. We also include an open-ended question for suggestions and comments. The VAS scale will be sent on WhatsApp as a Google Drive link. This same questionnaire has been administered by other authors^22,23^. If the participants do not complete the questionnaire within 45 days, it will be administered through a phone interview with the participants’ caregivers.

*Discomfort*

Patient discomfort during consultation will be assessed by the number of consultations with internet connection issues and/or the interviewer’s perception of patient behavior changes divided by the number of consultations.

*Travel time and cost savings*

Information on travel time and costs will be collected during the interview to capture both time and financial burden of face-to-face consultations. They will be asked about transportation, average travel time from the clinic to their home, and travel expenses.

*Statistical analysis*

For numerical variables, data will be presented as means, standard deviations and medians. For categorical variables, data will be described as frequencies.

Data Collection Methods

The team will be trained and will meet before the trial onset to consolidate data collection procedures.

Data Mangement

The study data will be collected and managed using the REDCap electronic data collection and management tool hosted at the Clinical Research Unit of the UFC.

The original study forms will be entered in REDCap.

ETHICS AND DISSEMINATION

We are committed to disseminating the methods and conclusions of the TeleGeriatrics Study to the interested research groups. Therefore, our disclosure plan after completion of the study covers the following: scientific meeting with residents, students, assistant doctors and researchers of HUWC in which the present study design, results and interpretation will be presented.

CONSENT OR ASSENT

The recruiting medical team will present the study details to the patients and will apply the consent form. The recruitment team will encourage patients put forth their doubts and assure them that their privacy will be preserved, and they are free to withdraw from the study anytime.

CONFIDENTIALITY

All information of the study patients will be stored in a locker in an area with limited access. The database will be registered and stored in REDCap, with password protected access systems.

DECLARATION OF INTERESTS

The author(s) received no specific funding for this work. There is no conflict of interests.

ACCESS TO DATA

The project data sets will be hosted on the REDCap, to which the project’s principal investigators will have direct access through a valid password.

ANCILLARY AND POST-TRIAL CARE

The study patients will continue to be assisted at the movement disorders outpatient clinic of HUWC by a team of neurologists and geriatricians.

DISSEMINATION POLICY: TRIAL RESULTS

Our purpose is to dedicate our efforts to reduce the interval between the completion of data collection and the dissemination of study results to an absolute minimum. We expect to take about 6 months to prepare the document of final results for an appropriate journal. The results of the study will be released to participating physicians, referring doctors, patients and the medical community.

|  |
| --- |

Figure 1. CONSORT 2010 Flowchart

Eligible

Assessment for eligibility

Excluded

## Baseline assessment

Allocation

Received virtual consultation

## Post-intervention Satisfaction

## Survey

Lost to follow-up

## Analysis

**References**

1. Governo do Estado do Ceará. *Decreto N^o^ 33.519, de 19 de Março de 2020*.; 2020.

2. BR. Lei n^o^ 13.979. *Diário Of da União*. 2020:2020.

3. Neumann LTV, Albert SM. Aging in Brazil. *Gerontologist*. 2018;58(4):611-617. doi:10.1093/geront/gny019

4. Schmidt MI, Duncan BB, E Silva GA, et al. Chronic non-communicable diseases in Brazil: Burden and current challenges. *Lancet*. 2011;377(9781):1949-1961. doi:10.1016/S0140-6736(11)60135-9

5. Phan S V., Osae S, Morgan JC, Inyang M, Fagan SC. Neuropsychiatric Symptoms in Dementia: Considerations for Pharmacotherapy in the USA. *Drugs R D*. 2019;19(2):93-115. doi:10.1007/s40268-019-0272-1

6. Tonelli M, Wiebe N, Straus S, et al. Multimorbidity, dementia and health care in older people:a population-based cohort study. *C Open*. 2017;5(3):E623-E631. doi:10.9778/cmajo.20170052

7. Bradford NK, Caffery LJ, Smith AC. Correction: Telehealth services in rural and remote Australia: A systematic review of models of care and factors influencing success and sustainability [Rural and Remote Health, 16, (2016) 4268]. *Rural Remote Health*. 2016;16(4):1-23.

8. Maldonado JMS de V, Marques AB, Cruz A. Telemedicine: challenges to dissemination in Brazil. *Cad Saude Publica*. 2016;32(suppl 2):1-11. doi:10.1590/0102-311x00155615

9. Emmerson LC, Ben-Zeev D, Granholm E, Tiffany M, Golshan S, Jeste D V. Prevalence and longitudinal stability of negative symptoms in healthy participants. *Int J Geriatr Psychiatry*. 2009;24(12):1438-1444. doi:10.1002/gps.2284

10. Monaghesh E, Hajizadeh A. The role of telehealth during COVID-19 outbreak: A systematic review based on current evidence. 2020;4:1-9. doi:10.21203/rs.3.rs-23906/v1

11. Chan AW, Tetzlaff JM, Gøtzsche PC, et al. SPIRIT 2013 explanation and elaboration: guidance for protocols of clinical trials. *BMJ*. 2013;346. doi:10.1136/bmj.e7586

12. Giansanti D. WhatsApp in mHealth: an overview on the potentialities and the opportunities in medical imaging. *mHealth*. 2020;6(3):19-19. doi:10.21037/mhealth.2019.11.01

13. Harris PA, Taylor R, Minor BL, et al. The REDCap consortium : Building an international community of software platform partners. *J Biomed Inform*. 2019;95(May):103208. doi:10.1016/j.jbi.2019.103208

14. Reisberg B, Franssen EH, Souren LEM, Auer SR, Akram I, Kenowsky S. Evidence and mechanisms of retrogenesis in Alzheimer’s and other dementias: Management and treatment import. *Am J Alzheimers Dis Other Demen*. 2002;17(4):202-212. doi:10.1177/153331750201700411

15. Sclan SG, Ph D, Reisberg B. <FAST tool initial work.pdf>. *Int Psychogeriatrics*. 1992;4(1):55-69. doi:10.1017/s1041610292001157

16. Reisberg B, Jamil IA, Khan S, et al. Staging Dementia. *Princ Pract Geriatr Psychiatry Third Ed*. 2010:162-169. doi:10.1002/9780470669600.ch31

17. Swain DG, O’Brien AG, Nightingale PG. Cognitive assessment in elderly patients admitted to hospital: The relationship between the shortened version of the abbreviated mental test and the abbreviated mental test and mini-mental state examination. *Clin Rehabil*. 2000;14(6):608-610. doi:10.1191/0269215500cr368oa

18. Ritter SRF, Zoccoli TLV, Lins MMP, Cardoso AF, Freitas MPD, Camargos EF. Adaptação de teste para rastreio de delirium em idosos admitidos em serviço de urgência. *Geriatr Gerontol Aging*. 2018;12(2):81-88. doi:10.5327/z2447-211520181800032

19. Schofield I, Stott DJ, Tolson D, McFadyen A, Monaghan J, Nelson D. Screening for cognitive impairment in older people attending accident and emergency using the 4-item Abbreviated Mental Test. *Eur J Emerg Med*. 2010;17(6):340-342. doi:10.1097/MEJ.0b013e32833777ab

20. Lino VTS, Pereira SRM, Camacho LAB, Ribeiro Filho ST, Buksman S. Adaptação transcultural da Escala de Independência em Atividades da Vida Diária (Escala de Katz). *Cad Saude Publica*. 2008;24(1):103-112. doi:10.1590/s0102-311x2008000100010

21. Korn RE, Shukla AW, Katz M, et al. Virtual visits for Parkinson disease. *Neurol Clin Pract*. 2017;7(4):283-295. doi:10.1212/CPJ.0000000000000371

22. Monteiro EP, Leonardo A. Peyré-Tartaruga, Ana p. J. Zanardi, Edson S. da Silva, Pedro Jimezes-Reyes, Jean-Benoit Morin ASP. Sprint exercise for subjects with mild-to-moderate Parkinson’s disease: feasibility and biomechanical outputs Abbreviated. *Clin Biomech*. 2019;72(2):69-76. doi:10.1016/j.clinbiomech.2019.11.012

23. Brokelman RBG, Haverkamp D, van Loon C, Hol A, van Kampen A, Veth R. The validation of the visual analogue scale for patient satisfaction after total hip arthroplasty. *Eur Orthop Traumatol*. 2012;3(2):101-105. doi:10.1007/s12570-012-0100-3
